# Microbiota Features Associated With a High-Fat/Low-Fiber Diet in Healthy Adults

**DOI:** 10.3389/fnut.2020.583608

**Published:** 2020-12-18

**Authors:** María Bailén, Carlo Bressa, Sara Martínez-López, Rocío González-Soltero, Maria Gregoria Montalvo Lominchar, Celia San Juan, Mar Larrosa

**Affiliations:** MAS Microbiota Group, School of Biomedical and Health Sciences, Universidad Europea de Madrid, Madrid, Spain

**Keywords:** microbiota, saturated-fatty acids, fiber, obesity, inflammation

## Abstract

A high intake of dietary saturated fatty acids (SFAs) is related to an increased risk of obesity, inflammation and cancer-related diseases, and this risk is attenuated only when SFAs are replaced by unsaturated fats and unrefined carbohydrates. The gut microbiota has recently emerged as a new environmental factor in the pathophysiology of these disorders, and is also one of the factors most influenced by diet. We sought to determine whether the gut microbiota of healthy individuals whose intake of SFAs exceeds World Health Organization (WHO) recommendations exhibits features similar to those reported in people with obesity, inflammation, cancer or metabolic disease. Healthy non-obese subjects were divided into two groups based on their SFAs intake. Body composition and gut microbiota composition were analyzed, and associations between bacterial taxa, diet and body fat composition were determined globally and separately by sex. Metagenome functional pathways were predicted by PICRUSt analysis. Subjects whose SFAs intake exceeded WHO recommendations also had a dietary pattern of low fiber intake. This high saturated fat/low fiber diet was associated with a greater sequence abundance of the *Anaerotruncus* genus, a butyrate producer associated with obesity. Analysis of data of high SFAs intake by sex showed that females presented with a greater abundance of *Campylobacter, Blautia, Flavonifractor* and *Erysipelatoclostridium*, whereas males showed higher levels of *Anaerotruncus, Eisenbergiella*, a genus from the order *Clostridiales* (*FamilyXIIIUCG_001)* and two genera from the *Lachnospiraceae* family. PICRUSt analysis confirmed these data, showing a correlation with a decrease in the abundance of sequences encoding for transporters of some metals such as iron, which is needed to maintain a healthy metabolism. Thus, the microbiota of healthy people on a high SFAs diet contain bacterial taxa (*Anaerotruncus, Lachnospiraceae Flavonifractor, Campylobacter, Erysipelotrichacea and Eisenbergiella*) that could be related to the development of some diseases, especially obesity and other pro-inflammatory diseases in women. In summary, the present study identifies bacterial taxa that could be considered as early predictors for the onset of different diseases in healthy subjects. Also, sex differences in gut microbiota suggest that women and men differentially benefit from following a specific diet.

## Introduction

An unbalanced or unhealthy diet is associated with the onset of several diseases. For example, a diet rich in red meat has been linked to colorectal cancer, and there is strong evidence for the involvement of some dietary fats in weight increase and cardiovascular disease (CVD) (1). The increased intake of saturated fatty acids (SFAs) is associated with higher levels of low-density lipoprotein (LDL) in serum (2) and elevated oxidative stress, which can play a significant role in cancer progression and colonic inflammation (3). There is also evidence to suggest that decreasing the intake of SFAs and replacing them with simple carbohydrates does not reduce CVD incidence, and only when SFAs are replaced by unsaturated fats (mainly polyunsaturated) is a decrease in CVD incidence observed (4). The World Health Organization (WHO) recommends that 30% or less of the total energy intake in adults should come from dietary lipids, with SFAs comprising <10% and *trans*-fats <1% (5, 6). However, the last few decades have seen a significant increase in the consumption of processed and ultra-processed food products, which are rich in fats, particularly SFAs. Indeed, a recent cross-sectional study performed in a nationally representative Spanish population found that total fat intake exceeded 38% of the total energy intake, with SFAs accounting for over 11.5% (7). Moreover, a high-fat diet (HFD) in humans is associated with a reduced consumption of carbohydrates, especially those that constitute the dietary fiber, and the average fiber intake rarely reaches the daily recommendation of >25 g/d (8).

Accumulating data suggest that the relationship between our diet and our health is mediated, at least in part, by the gut microbiota, which has recently emerged as an influential factor in many diseases. Imbalances in the gut microbiota (termed dysbiosis) are associated with colon cancer, metabolic syndrome, diabetes, and cardiovascular events, among others (9). Among the known factors that can shape the gut microbiota, dietary components are one of the most important (10), and several studies have shown that HFD modifies the gut microbiota of both animals and humans. For example, HFD-fed mice show increases in the abundance of *Firmicutes, Proteobacteria and Actinobacteria*, and concomitant reductions in health-promoting bacteria such as those of the *Bacteroidetes* phylum, and *Bifidobacterium* and *Akkermansia* genera (11–15). Additionally, HFD diminishes microbial richness and increases the abundance of Gram-negative bacteria, which can enhance intestinal permeability (16). In turn, this can lead to the translocation of lipopolysaccharide (LPS) to the systemic circulation (17), triggering low-grade inflammation (18, 19). HFD consumption in humans results in loss of bacterial diversity (20), with a higher abundance of *Bilophila, Alistipes, Blautia* and several genera of the *Gammaproteobacteria* class, and a lower abundance of *Bacteroides, Clostridium*, and *Roseburia* spp. (21, 22). Importantly, not only is the quantity of fat important, but also the type. Animal studies indicate that whereas SFAs have a detrimental effect on gut microbiota, weight gain, intestinal permeability and proinflammatory status, monounsaturated and polyunsaturated fats can have beneficial effects on the host microbial ecosystem, body weight loss and anti-inflammatory response (23). To date, however, very few studies have explored the relationship between SFAs intake and gut microbiota. In light of the above evidence, the present study was designed to compare the microbiota features of subjects whose SFAs intake exceeded WHO recommendations with those who consumed recommended amounts of SFAs. We studied the microbiota composition of healthy non-obese men and women to explore features that could precede the development of some chronic disorders in a healthy population. We analyzed all data by sex, as the parameters of body composition, dietary requirements predisposition, incidence of diseases and associated microbiota dysbiosis differs between men and women (24).

## Materials and Methods

### Study Design and Subjects

This was an observational cross-sectional study of healthy individuals. Volunteers were recruited using posters, social networks, and magazines. The study was carried out at Universidad Europea de Madrid (Madrid, Spain), and was limited to men and women aged 18–45 years and with a body mass index (BMI) of 18.5–25 kg/m^2^. Exclusion criteria were the following: any kind of pathology (during the study or in the 6 months prior to it), previous gastrointestinal surgery, antibiotics intake or modification of diet during 3 months prior to the study, intake of any drug (with the exception of contraceptive pills) 1 month before the study, smoking, use of prebiotics or probiotics, vegetarian or vegan diets, nutritional or ergogenic complements, pregnancy, or lactation. All subjects were Caucasian. A total of 109 volunteers were recruited and gave written and informed consent prior to participation. According to WHO recommendations on SFAs intake, which should be <10% of total energy intake, volunteers were divided into two groups based on the results of a validated food frequency questionnaire (FFQ): low SFA intake (LSFA), when intake was <9% of total energy, and high SFA intake (HSFA) when is more than 11% of total energy (6). Volunteers with intermediate (9–11%) intake of SFAs were not considered for the study (Supplementary Figure 1). The Ethics Committee for Clinical Research of the Hospital Ramón y Cajal (Madrid, Spain) approved the study (protocol #CEIC 338-17).

### Anthropometry and Body Composition

Height and weight were measured with a stadiometer (Asimed T2, Barcelona, Spain) and a balance scale (Año Sayol SL, Barcelona, Spain), respectively; and body mass index (BMI) was calculated as weight (kg) divided by height (m^2^). Body composition was evaluated on the day of sample collection by dual-energy X ray absorptiometry (DEXA) (Hologic DEXA scan, Hologic Inc, Barcelona, Spain). The following body composition measurements were collected: estimated visceral adipose tissue (VAT), body fat percentage (BFP), body fat mass (BFM), total lean mass and fat and lean mass distribution in the trunk and extremities. The following indices were calculated using the obtained values: adiposity index (AI) = total fat/height^2^; lean mass index (LMI) = total lean mass/height^2^; and appendicular lean mass index (AppLMI) = lean mass in arms + legs/height^2^.

### Stool Collection and Bacterial DNA Extraction

Participants were provided with an Fe-Col® Fecal Sample Collection Kit (Alpha Laboratories Ltd., Eastleigh, Hampshire, UK) and an icebox and a cooler to maintain the samples at −20°C until they reached the laboratory where they were aliquoted and conserved at −80°C. Bacterial DNA was extracted using the E.Z.N.A.® Stool DNA Kit (Omega-Biotek, Norcross, GA) and a bead-beating homogenizer (Bullet Blender Storm, Next Advance, Averill Park, NY). Concentration and purity of DNA were measured on a Nanodrop 1000 spectrophotometer (ThermoFisher Scientific, Waltham, MA).

### Food Frequency Questionnaire

Dietary pattern assessment was carried out using a validated FFQ with 93 food items (25). The FFQ was given to participants to complete on the day they donated the fecal samples. Data from the FFQ were analyzed using Dietsource software 3.0 (Novartis, Barcelona, Spain) to obtain the total amount of carbohydrates, protein, fats and fiber ingested, and the total energy ingested.

### Sequencing and Bioinformatics

The hypervariable regions V3 and V4 of the bacterial 16S rRNA gene were amplified using the primer pair 5′-TCGTCGGCAGCGTCAGATGTGTATAAGAGACAG-3′ and 5′ GTCTCGTGGGCTCGGAGATGTGTATAAGAGACAG-3′ (26). The 459-bp amplicon was visualized in an ethidium bromide-stained 0.8% agarose gel, and bands were excised and cleaned using the MinElute Gel Extraction Kit (Qiagen, Hilden, Germany). DNA amplicons were sequenced on a MiSeq Illumina platform (Illumina, San Diego, CA). Sequence outputs were analyzed using the Quantitative Insights into Microbial Ecology (QIIME2) program v18.2, April 2018 (27). The 16S paired reads were imported in QIIME2 and processed with the DADA2 plugin (28), adjusting the maximum expected error threshold to 1.0 (29) and filtering chimera with the consensus method. Taxonomy classification was assigned with the q2-feature-classifier plugin (30) using the BLAST+ method (31) with the following settings: reference sequences SILVA (32, 33) database release 132 clustered at 97% sequence similarity; reference taxonomy label SILVA taxonomy 7 levels; maximum number of hits to keep for each query, 1; reject match if percent identity to query is lower than 0.9. Diversity analyses were conducted using the q2-diversity plugin in QIIME2. Beta-diversity was evaluated by calculating Bray-Curtis, Jaccard, unweighted and weighted Unifrac distance metrics (34). To study alpha diversity, observed operational taxonomic units (OTUs), evenness, and Shannon and Faith's phylogenetic diversity indices were calculated.

Kyoto Encyclopedia of Genes and Genomes (KEGG) ortholog abundance predictions were obtained with PICRUSt2 (35) software (Phylogenetic Investigation of Communities by Reconstruction of Unobserved States) using the default “max parsimony” method for hidden-state prediction and a NSTI (Nearest Sequenced Taxon Index) value of 0.1.

### Statistical Analysis

Statistical analysis was carried out using QIIME2 v18.2, SPSS software 22.0 (SPSS, Chicago, IL) and the R statistical package 3.5.2. Variable normal distribution was assessed using the Shapiro-Wilk test. When normal distribution was not assumed, non-parametric tests were performed. Intergroup comparisons of variables were performed with a *t*-test or the Kruskal-Wallis test. Linear discriminant analysis coupled with effect size (LEfSe v1.0) was performed to identify bacterial taxa differentially represented between groups at species or higher taxonomy levels (36). Significance was set initially at *p* < 0.05 and when correlation analyses were performed, data were corrected with the Benjamini-Hochberg false discovery rate (FDR); FDR-adjusted *p*-values or *q*-values < 0.3 were considered significant. Linear regression analysis was performed for those microbial taxa that were significantly different between high and low SFAs consumption. The stepwise backward elimination method was used with the covariates that were significantly different between groups, considering diet variables (carbohydrates, protein, fats, SFAs, mono- and polyunsaturated fatty acids [MUFAs and PUFAs, respectively], ratio SFAs/unsaturated fat and fiber). Multiple regression models were built when multiple variables were predictive.

## Results

### Subjects and Dietary Habits

The study design flow-chart is shown in Supplementary Figure 1. Of the 109 volunteers recruited, 71 met the inclusion criteria and included 37 men and 34 women (S1 aged between 22 and 43 years. Among the men, 17 followed a diet low in SFAs (LSFA-M, SFA (%) = 7.49 ± 1.0) and 20 followed a diet high in SFAs (HSFA-M, SFA (%) = 12.62 ± 1.30). Among the women, 18 followed a diet low in SFAs (LSFA-W, SFA (%) = 7.12 ± 1.31) and 16 a diet high in SFAs (HSFA-W, SFA (%) = 12.25 ± 1.01) (Table 1).

**Table 1:** Energy and dietary intake of participants.

	**LSFA**	**HSFA**	***P***	**LSFA-M**	**HSFA-M**	***p***	**LSFA-W**	**HSFA-W**	***p***
Energy (kcal/day)	2,273.95 ± 651.55	2,123.09 ± 756.48	0.368	2,060.48 ± 583.78	2,317.04 ± 903.44	0.32	2,466.99 ± 683.27	1,880.66 ± 434.26	**0.01**
Carbohydrates (%)	51.16 ± 5.79	41.61 ± 4.79	*p* < 0.001	51.47 ± 5.36	41.20 ± 4.61	**0.00**	50.95 ± 6.46	42.12 ± 5.11	**0.00**
Protein (%)	17.55 ± 2.89	16.89 ± 3.14	0.352	17.59 ± 2.76	17.60 ± 3.60	0.99	17.56 ± 3.17	16.00 ± 2.25	0.11
Fat (%)	31.25 ± 5.79	41.41 ± 4.54	*p* < 0.001	30.94 ± 5.90	41.10 ± 4.94	**0.00**	31.5 ± 6.02	41.81 ± 4.10	**0.00**
SFA (%)	7.29 ± 1.17	12.45 ± 1.18	*p* < 0.001	7.49 ± 1.05	12.62 ± 1.30	**0.00**	7.12 ± 1.31	12.25 ± 1.01	**0.00**
MUFA (%)	12.86 ± 3.19	17.97 ± 3.85	*p* < 0.001	13.20 ± 3.56	17.64 ± 4.15	**0.00**	12.51 ± 2.96	18.37 ± 3.53	**0.00**
PUFA (%)	3.26 ± 0.88	4.07 ± 0.93	*p* < 0.001	3.53 ± 0.94	3.90 ± 0.93	0.24	2.99 ± 0.78	4.28 ± 0.91	**0.00**
SFA/Unsaturated ratio	0.47 ± 0.09	0.59 ± 0.13	p < 0.001	0.46 ± 0.09	0.62 ± 0.149	**0.00**	0.47 ± 0.11	0.56 ± 0.10	**0.03**
Fiber (g/day)	29.06 ± 12.39	18.14 ± 7.27	p < 0.001	27.55 ± 14.16	18.77 ± 7.90	**0.02**	30.43 ± 11.09	17.34 ± 6.56	**0.00**

*Values are mean ± standard deviation. LSFA-M, men with low saturated fatty acid intake; HSFA-M, men with high saturated fatty acid intake; LSFA-W, women with low saturated fatty acid intake; HSFA-W, women with high saturated fatty acid intake; SFA, saturated fatty acids; MUFA, monounsaturated fatty acids; PUFA, polyunsaturated fatty acids; Unsaturated Fat comprises the sum of MUFA + PUFA; Bold values: p < 0.05*.

Data on macronutrients and food intake extracted from the FFQ are shown in Table 1 and Supplementary Table 1, respectively. No significant differences between the groups were found for energy or protein intake. Dietary reports of the LSFA groups (both men and women) revealed a balanced diet for carbohydrates and fats, in accordance with recommendations for 50–60% carbohydrates and 30–35% fat, although protein intake was above the recommended dose (10–15% proteins). Dietary reports of the HSFA groups revealed that total fat intake was above the recommended dose and carbohydrates intake was below (Table 1). Fiber intake was significantly lower in the HSFA group than in the LSFA group (*p* < 0.01) and was below WHO recommendations (25 g/d). Likewise, the consumption of vegetables, fruits, and nuts was significantly lower in the HSFA group than in the LSFA group, whereas the consumption of potatoes was significantly higher (Supplementary Table 1). Correlation analyses of dietary variables showed a positive association between intake of saturated fats and intake of total fat (*R* = 0.866, *q* < 0.001), MUFAs (*R* = 0.792, *q* < 0.001), PUFAs (*R* = 0.755, *q* < 0.001), foods containing saturated fats (dairy products, *R* = 0.370, *q* = 0.004; chocolate, *R* = 0.379, *q* = 0.02; and red meat, *R* = 0.493, *q* < 0.001), and also potatoes (*R* = 0.359, *q* = 0.004), potato chips (*R* = 0.571, *q* = 0.007), and white bread (*R* = 0.359, *q* = 0.001). Saturated fat intake was negatively associated with the intake of fiber (*R* = −0.503, *q* < 0.001), fruits (*R* = −0.489, *q* < 0.001), vegetables (*R* = −0.313, *q* = 0.008), and nuts (*R* = −0.279, *q* = 0.03).

Dietary habits were also analyzed by sex. Total fat consumption was significantly higher (double) in the HSFA-M (male) group than in the LSFA-M group (*p* < 0.01). Likewise, the intake of MUFAs was significantly higher in the HSFA-M group, whereas no differences were found for PUFAs intake (Table 1). Analysis of food consumption showed a significantly higher intake of potatoes, red meat and processed meat in the HSFA-M group than in the LSFA-M group (Supplementary Table 1), whereas the opposite was seen for fruit intake (Supplementary Table 1). For women, significant differences were found in the energy provided by diet (*p* = 0.01), which was higher in the LSFA-W group than in the HSFA-W group. The LSFA-W group also had a higher intake of carbohydrates (*p* < 0.001) and fiber (*p* < 0.001). Differences were also found in relation to the percentages of the energy provided by total fat and the percentages of the different types of fat, with the consumption of all being significantly greater in women following an HSFA diet (Table 1). In contrast to the results for men, no differences were found in the consumption of red and processed meat in the women SFA groups, but the intake of fruits and vegetables and fish were significantly lower in the HSFA-W group than in the LFSA-W group (*p* = 0.03) (Supplementary Table 1).

As body composition differs between in men and women, body composition data were stratified for sex. While no significant differences in body mass were found between groups, BMI was significantly different between the HSFA-M and LSFA-M groups (Supplementary Table 2). Parameters related to body fat composition, BFP, VAT and AI, were all significantly higher in the HSFA-M group, whereas no differences were found in relation to lean mass. No significant differences were found in the body composition parameters of women (Supplementary Table 2).

### Fecal Microbiota

Analysis of the sequencing data showed that average number of reads per sample was 26,890. Rarefaction curves based on observed species, Shannon, and phylogenetic distance measures were virtually saturated, indicating sufficient sequencing depth (data not shown). There were no significant differences in alpha-diversity parameters between the LSFA and HSFA groups: observed OTUs (*p* = 0.872), Shannon (*p* = 0.279), evenness (*p* = 0.052) or Faith's phylogenetic diversity (*p* = 0.774). There was, however, a trend toward significance for evenness, which would indicate probable differences in taxa abundance distribution between the groups. Also, no significant differences were observed for beta-diversity (Bray-Curtis *p* = 0.139; Jaccard *p* = 0.137; unweighted *p* = 0.215 and weighted Unifrac *p* = 0.741, distance metrics). When data were analyzed by sex, no significant differences between groups (HSFA-M, LSFA-M, HSFA-W and LSFA-W) were found for alpha- or beta-diversity parameters. Likewise, no significant differences were found when the ratio *Firmicutes*/*Bacteroidetes* (F/B ratio) of the HSFA and LSFA groups were compared (*p* = 0.639), or when data were analyzed by sex (HSFA-M F/B ratio = 0.60 ± 0.33, LSFA-M F/B ratio = 0.79 ± 0.33 *p* = 0.120; HSFA-W F/B ratio = 1.27 ± 0.63, LSFA-W F/B ratio = 1.32 ± 0.77 *p* = 0.866). Significant associations were found for the entire population when the F/B ratio was considered: a positive association was found between this ratio and the total percentage of body fat (*R* = 0.389, *p* = 0.02), and a negative association was found for body composition lean mass (*R* = −0.429, *p* = 0.001).

**Figure 1:**
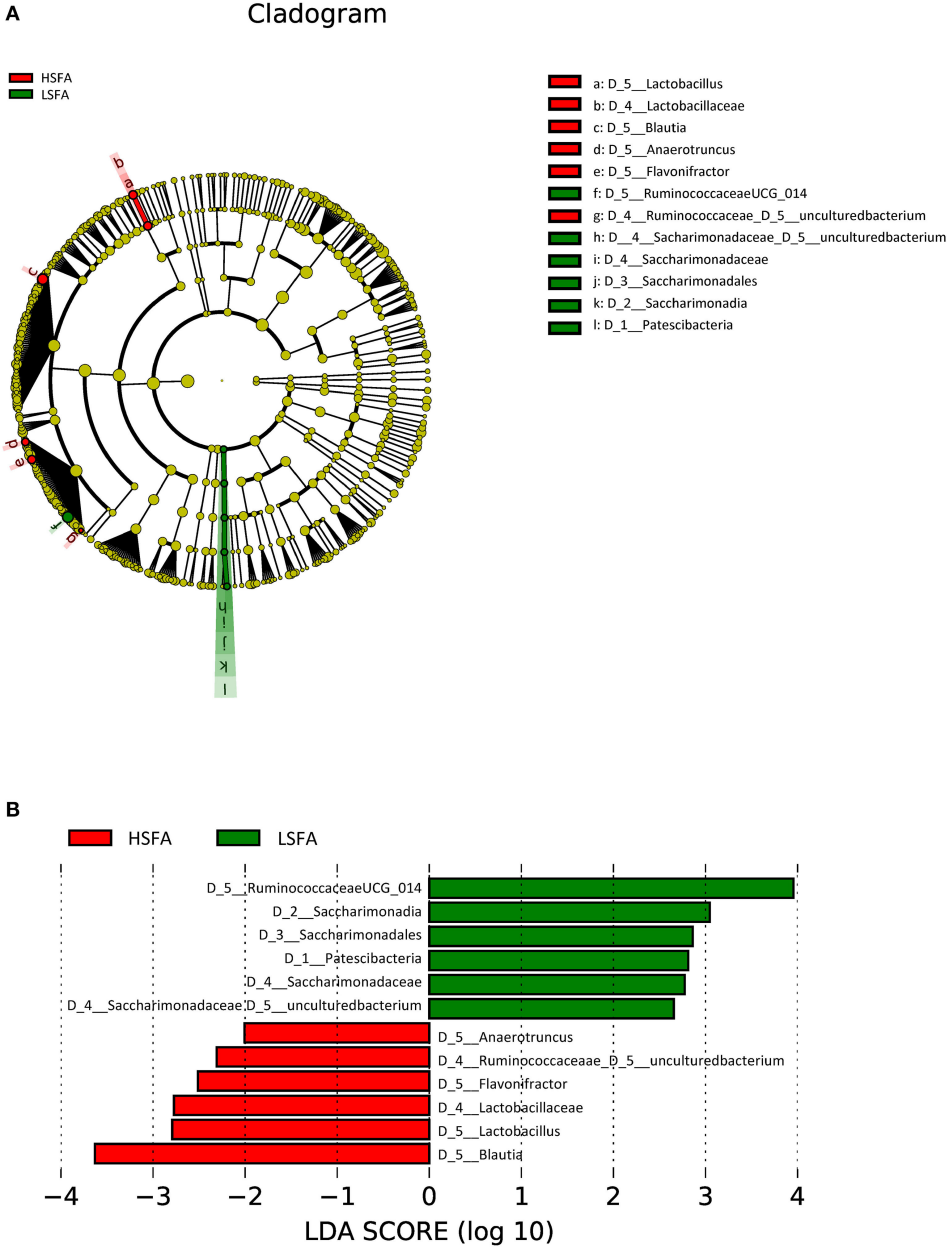
Cladogram **(A)** and histogram **(B)** representation of differentially abundant genera between LSFA and HSFA groups. The central point represents the root of the tree (*Bacteria*), and each ring represents the next lower taxonomic level (phylum through genera). The diameter of each circle represents the relative abundance of the taxon; Histogram of LDA score, only the taxa with a significant LDA threshold value above 2 are shown.

LEfSe analysis of the entire population studied (considering both men and women) at the genus level showed an increase in the abundance of sequences belonging to *Blautia, Lactobacillus, Flavonifractor, Anaerotruncus* genera and an uncultured genus of the *Ruminoccococeae* family in the HSFA group, whereas the microbiota of the LSFA group was enriched for a genus from the *Ruminococcaceae* family (*Ruminococcaceae* UCG014), *Saccharimonadales, Patescibacteria* and an uncultured genus of the *Saccharimonadaceae* family (Figure 1).

**Figure 2:**
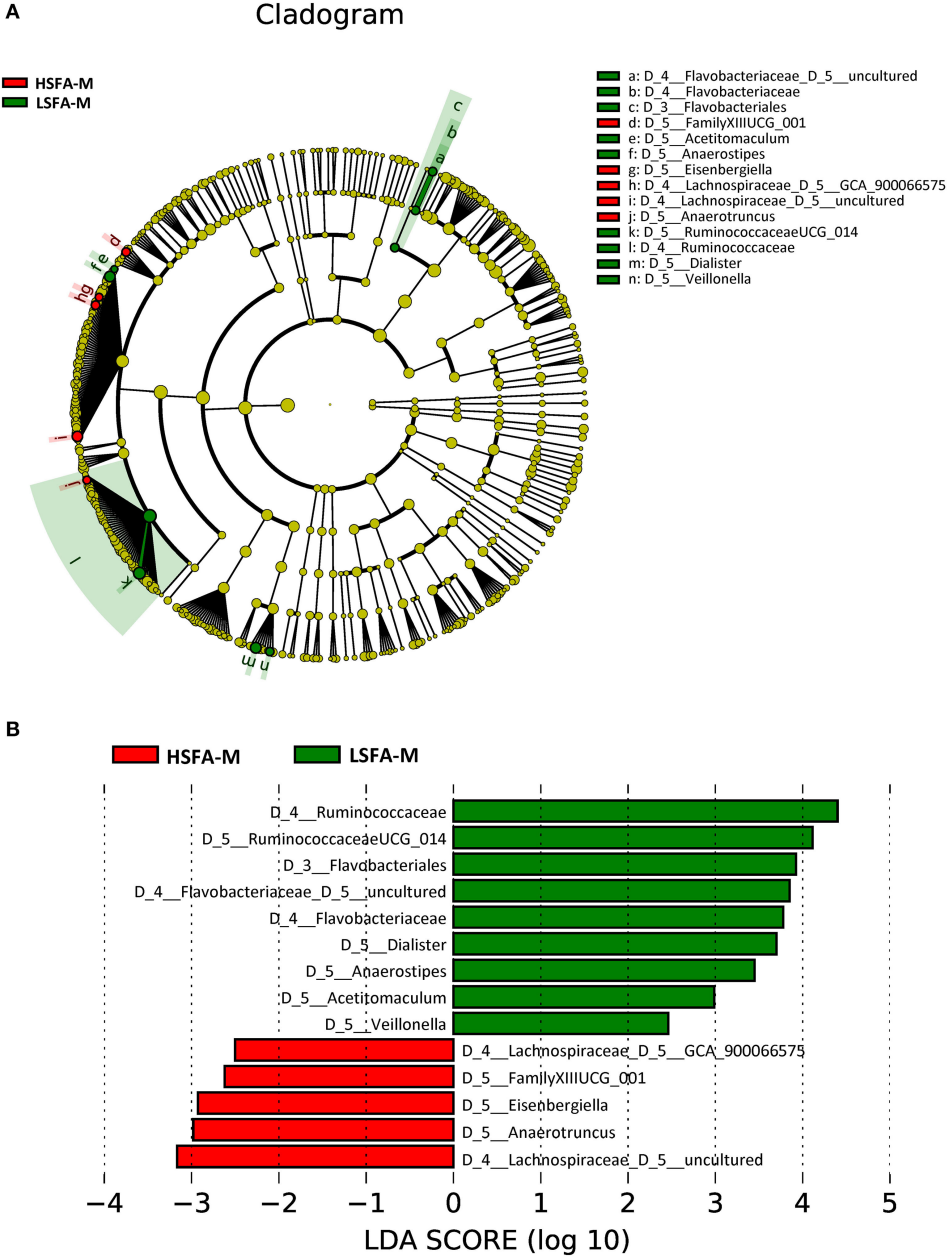
Cladogram **(A)** and histogram **(B)** representation of differentially abundant genera in men, between LSFA-M and HSFA-M groups.

Analysis by sex revealed that the predominant taxa for the HSFA-M group included an uncultured genus from the *Lachnospiraceae* family, the genera *Anaerotruncus, Eisenbergiella*, a genus from the order *Clostridiales* (*FamilyXIIIUCG*_001), and the *GCA_900066575* genus from the *Lachnospiraceae* family, whereas for the LSFA-M group the preponderant taxa at the genus level were a genus from the *Ruminococcaceae* family (*Ruminococcaceae UCG_014*), an uncultured genus of the *Flavobacteriaceae* family, and *Dialister, Anaerostipes, Acetitomaculum*, and *Veillonella* genera (Figure 2A). The highest LDA score for the HSFA-M group was an unidentified genus of the *Lachnospiraceae* family, whereas the highest score in the LSFA-M group was for an unknown genus of the *Ruminococcaceae* family (Figure 2B).

**Figure 3:**
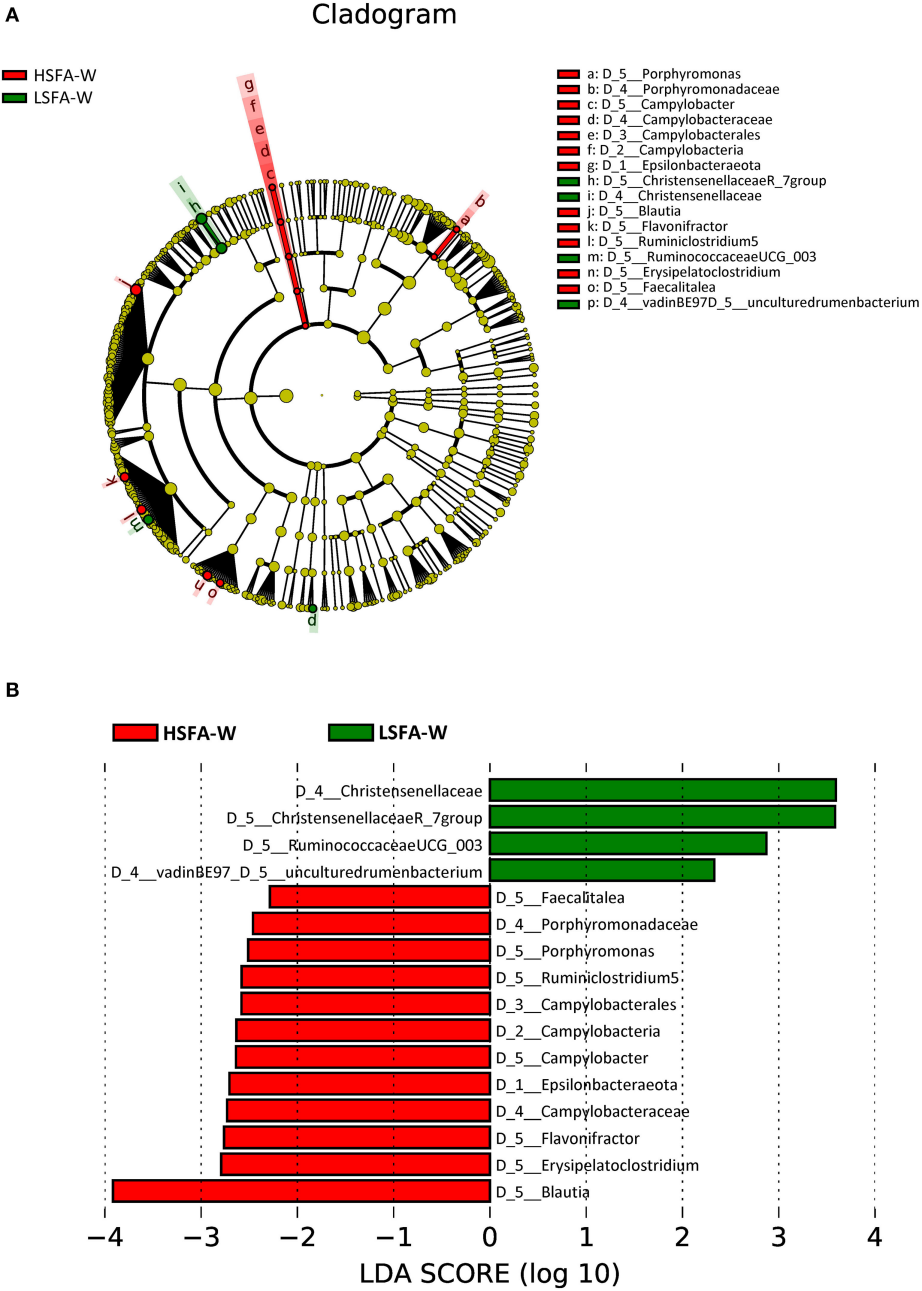
Cladogram **(A)** and histogram **(B)** representation of differentially abundant genera in women between LSFA-W and HSFA-W groups.

Analysis of the microbiota from the HSFA-W group revealed an enrichment of the phylum *Epsilobacteraeota*, specifically the *Campylobacter* genus. In addition, there was a higher representation of *Blautia, Erysipelatoclostridium, Flavonifractor*, a genus from the *Ruminococcaceae* family (*Ruminiclostridium 5), Porphyromonas*, and *Faecalitalea* in the HSFA-W group (Figure 3). By contrast, the LSFA-W group showed a greater abundance of *Christensenellaceae* (*R_*7) and *Ruminococcaceae* (*UCG_003*) genera, and an uncultured rumen bacterium of the *vadinBE97* family (Figure 3). The highest LDA scores were for *Blautia* in the HSFA-W group, and for an uncultured genus of the *Christensenellaceae* family in the LSFA-W group (Figure 3).

### Correlation of Gut Microbial Composition and Diet

**Figure 4:**
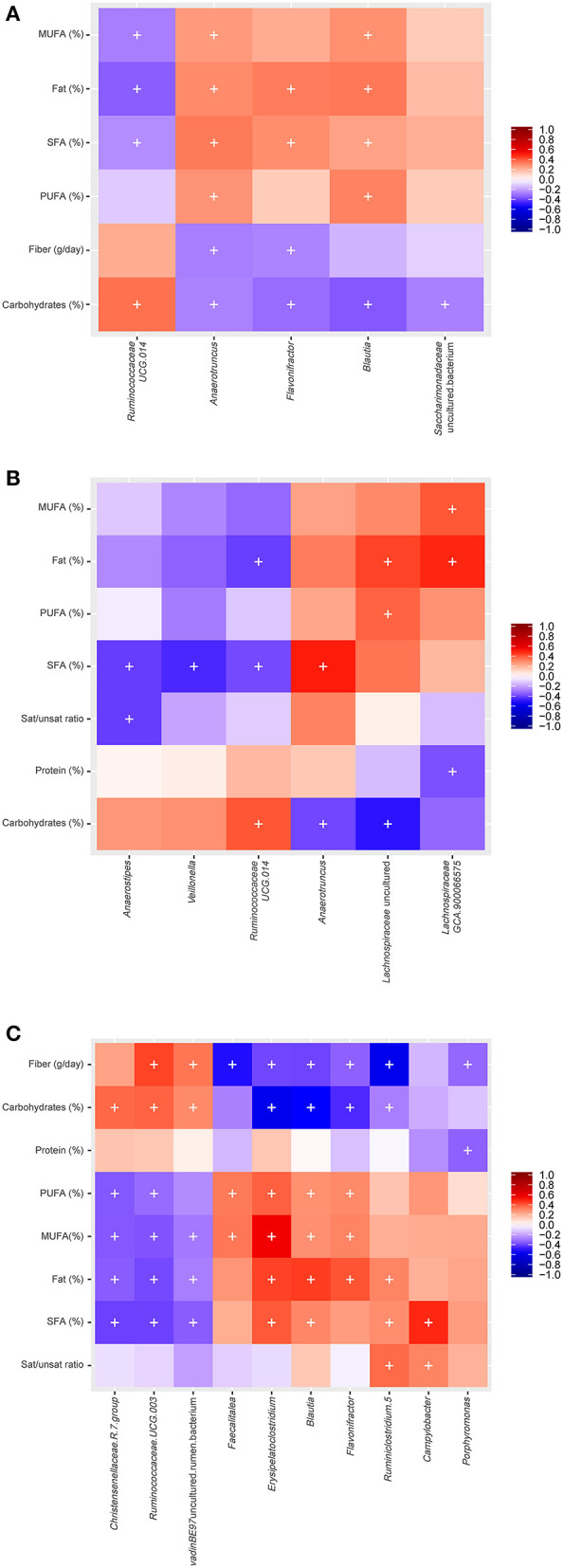
Spearman correlation between gut microbiota and diet for the studied groups: **(A)** Both men and women **(B)** men **(C)** women (+: *p* ≤ 0.05; *p*.adj < 0.3, BH correction).

Non-parametric bivariate correlation analysis was performed to study the association between diet and gut microbiota. Spearman's rank heatmap of gut microbiota and diet (Figure 4) showed significant correlations for six genera in men, 10 genera in women, and five genera when men and women were studied altogether, according to SFAs consumption. For the entire population studied, *Anaerotruncus, Flavonifractor* and *Blautia* correlated negatively with carbohydrates and positively with total fat and SFAs, with the opposite found for *Ruminococcaceae* (*UCG.014*) (Figure 4A). For men, SFAs consumption correlated negatively with *Anaerostipes, Veillonella, Ruminococcaceae* (*UCG.014*) and positively with *Anaerotruncus* (Figure 4B). Moreover, positive correlations were found in women between fat (PUFAs, MUFAs, SFAs and total fat) and the genera *Faecalitalea, Erisipelatoclostridium, Blautia, Flavonifractor*, a genus from the *Ruminococcaceae* family (*Ruminiclostridium 5)* and *Campylobacter*, whereas negative correlations were found between fat and *Christensenellaceae* (*R_7* group), *Ruminococcaceae* (*UCG-00*), and an uncultured genus of *vadinBE97* (Figure 4C).

### Multiple Regression Analysis

Multiple regression analysis was performed to evaluate the influence of variables (diet and body composition) on differences found in microbial taxa (Tables 2–4). The presence of *Anaerotruncus* was positively explained by the amount of SFAs in diet, the abundance of *Blautia* was partly due to consumption of PUFAs, and the presence of *Flavonifractor* by total fats. Carbohydrates intake partially explained, either positively or negatively, the presence of *Ruminococcaceae* (*UCG014*), *Saccharimonadaceae* genus, *Flavonifractor*, and *Blautia* in the HSFA and LSFA groups (Table 2). For men, *Anaerotruncus* was positively and partially explained by SFAs, whereas *Dialister* and *Anaerostipes* were negatively explained by SFAs, and *Flavobacteriaceae* by red meat. The intake of carbohydrates was positively related to the presence of *Ruminococcaceae* (*UCG014*) and *Dialister*, the latter also being linked to the intake of fiber and fruits. The related *Ruminococcaceae* bacterial taxa enriched in the HSFA-M group were explained by MUFAs intake. Adiposity body parameters partially predicted the presence of *Veillonella* and *Eisenbergiella* and *Lachnospiraceae (GCA-900066575)* (Table 3). For women, total energy intake and fiber had a positive impact on *Ruminococcaceae* (*UCG-003*) genus and the *vadinBE97.5* family, whereas carbohydrates had a negative relation with *Flavonifractor, Blautia* and *Erysipelatoclostridium*. SFAs explained, in part, the presence of *Campylobacter*, whereas the differences in *Blautia, Flavonifractor* and *Erysipelatoclostridium* were explained by the amount of total fats and the intake of MUFAs and/or PUFAs (Table 4).

**Table 2:** Multivariate linear regression analysis of the bacterial taxa significantly different between HSFA and LSFA groups as dependent variants, and diet and body composition parameters as independent variants.

**Dependent variables**	**Independent variables**	***B***	***p***	***R^**2**^***
*Lactobacillus*	None			
*Anaerotruncus*	SFA (%)	0.252	0.034	0.063
*Ruminococcaceae UCG-014*	Carbohydrates (%)	0.304	0.010	0.092
	Fat (%)	−0.0306	0.009	0.094
*Flavonifractor*	Carbohydrates (%)	−0.0335	0.004	0.112
	Fat (%)	0.313	0.008	0.098
*Blautia*	Carbohydrates (%)	−0.335	0.004	0.112
	Fat (%)	0.313	0.008	0.098
	PUFA (%)	0.291	0.014	0.085
*Saccharimonadaceae*;	Carbohydrates (%)	0.238	0.046	0.057
uncultured bacterium	Fat (%)	−0.255	0.032	0.065
*Ruminococcaceae*;	None			
uncultured bacterium				

*B, regression coefficient*.

**Table 3:** Multivariate linear regression analysis of the bacterial taxa significantly different between HSFA-M and LSFA-M groups as dependent variants, and diet and body composition parameters as independent variants.

**Dependent variables**	**Independent variables**	***B***	***p***	***R^**2**^***
*Acetitomaculum*	None			
*Anaerotruncus*	SFA (%)	0.328	0.048	0.108
*Anaerostipes*	SFA (%)	−0.353	0.032	0.124
*Eisenbergiella*	BFP	0.367	0.030	0.135
	AI	0.380	0.024	0.145
*Lachnospiraceae GCA-900066575*	BMI	−0.410	0.008	0.344
	MUFA (%)	0.479	0.002	
*Lachnospiraceae* uncultured	Carbohydrates (%)	−0.375	0.022	0.141
	Fat (%)	0.400	0.014	0.160
	MUFA (%)	0.430	0.008	0.185
*Ruminococcaceae UCG-014*	Carbohydrates (%)	0.371	0.024	0.138
*Dialister*	SFA (%)	−0.403	0.013	0.162
	Carbohydrates (%)	0.346	0.036	0.120
	Fruits (s/d)	0.427	0.009	0.182
	Fiber (g)	0.390	0.017	0.152
*Flavobacteriaceae* uncultured	Red meat (s/w)	−0.371	0.026	0.138
*Veillonella*	BFP	−0.396	0.019	0.157
	AI	−0.343	0.044	0.118
*Family XIII UCG_001*	None			

*B, regression coefficient*.

**Table 4:** Multivariate linear regression analysis of the bacterial taxa significantly different between HSFA-W and LSFA-W groups as dependent variants, and diet and body composition parameters as independent variants.

**Dependent variables**	**Independent variables**	***B***	***p***	***R^**2**^***
*Porphyromonas*	None			
*Campylobacter*	SFA (%)	0.361	0.036	0.130
*Faecalitalea*	None			
*Christensenellaceae R_7group*	None			
*Ruminococcaceae UCG-003*	Energy (kcal)	0.382	0.026	0.146
	Fiber (g)	0.358	0.038	0.128
*vadinBE97* uncultured rumen bacterium	Energy (kcal)	0.553	0.001	0.305
	Fiber (g)	0.354	0.040	0.125
	Fish (s/w)	0.412	0.015	0.170
*Erysipelatoclostridium*	Carbohydrates (%)	−0.490	0.003	0.240
	Fat (%)	0.389	0.023	0.152
	MUFA (%)	0.447	0.008	0.200
	PUFA (%)	0.410	0.016	0.168
*Blautia*	Carbohydrates (%)	−0.491	0.003	0.241
	Fat (%)	0.442	0.009	0.195
	PUFA (%)	0.359	0.037	0.129
*Flavonifractor*	Carbohydrates (%)	−0.434	0.010	0.189
	Fat (%)	0.367	0.033	0.135
*Ruminiclostridium 5*	Fiber (g)	−0.419	0.014	0.175

*B, regression coefficient*.

### Predicted Functional Metagenome

PICRUSt analysis was used to infer the functional capabilities of the microbial communities by predicting functional genes that are associated with different taxa. LEfSe analysis identified several pathways that were enriched in high and low SFAs consumption groups. Considering the differences observed when analyzing microbiota composition, the PICRUSt and Lefse analysis were performed with the population stratified by sex.

Only the outer membrane receptor for ferrienterochelin and colicins (K16089) was enriched in HSFA-W (Supplementary Figure 2A). For men, there were differences in four pathways (response regulator HydG, L-fucose permease, other glycan degradation involving alpha-L-fucosidase and antimicrobial resistance with a multidrug efflux system) in the HSFA-M group, and only one in the LSFA-M group (ABC-2 type transport system ATP-binding protein) (Supplementary Figure 2B).

## Discussion

Unhealthy eating habits can lead to the development of numerous chronic illnesses such as metabolic syndrome, CVD, and diabetes. There is growing evidence that some of these disorders are associated with specific features of the gut microbiota, which differ from those of healthy individuals (37). However, it is not known with any precision when the microbiota begins to present characteristics that differ from those found in healthy subjects. We aimed to determine whether the microbiota of a healthy population, in the absence of obesity or any type of diagnosed disease, and following a diet rich in HSFA, has a higher proportion of bacterial taxa reported to be related to the onset of pathological processes. While the population was initially divided into low- and high-SFA intake according to dietary SFAs content, an intrinsic negative association between SFAs and fiber intake was observed. Therefore, our results show the effect of a diet rich in SFAs and low in fiber.

We analyzed the data by sex because of the known differences between males and females for predisposition and incidence of diseases, and development of microbiota dysbiosis (24). Our results indicate that the gut microbiota of both healthy men and women following an HSFA/low-fiber diet has a greater presence of bacteria that could be related to some chronic diseases, including *Anaerotruncus, Eisenbergiella, Lachnospiraceae, Campylobacter, Flavonifractor*, and *Erysipelatoclostridium*. High fat intake is considered an energy-dense diet and is usually associated with a low intake of carbohydrates, including fiber, and a moderate intake of proteins, which overall disrupts the dietary pattern and leads to an imbalanced diet (38). Obesity, diabetes and atherosclerosis have been previously associated with diets rich in fats but low in fiber (39). Dietary fiber contributes beneficially to gastrointestinal health and a “good” gut microbiota, whereas a deficiency in plant fibers, including legumes, grains, fruits and vegetables, is related to numerous gastrointestinal disorders. Although diet is known to shape the gut microbiota, there are relatively few human studies that address the effect of macronutrient content. It was initially thought that the interaction of dietary fats with intestinal microbiota was negligible, since dietary fat is absorbed in the proximal portion of the intestine and does not reach the colon. It is now known, however, that it does indeed reach the colon, where it can serve as a food substrate for some bacteria and also exert antimicrobial effects (23). Most studies focusing on the effect of HFD on gut microbiota have been performed in animal models (23), where it has been observed that HSFA diets induce microbial changes associated with obesity, including loss of bacterial diversity and inflammatory response (40).

We observed that when participants were studied together (both men and women), those with a high intake of SFAs (HSFA) showed an increased abundance of *Anaerotruncus, Eisenbergiella, Lachnospiraceae, Campylobacter, Flavonifractor*, and *Erysipelatoclostridium*, which are known to correlate with weight gain (41) and have also been reported in various diseases, such as obesity (42), colorectal cancer (43, 44), lupus erythematosus (45), allergies (40), and some intestinal diseases (46). Conversely, participants on an LSFA diet showed an increase in bacterial taxa linked to beneficial effects on health (47, 48).

Changes in the abundance of *Anaerotruncus, Anaerostipes*, and *Ruminococcaceae* (*UCG-014*) in men (HSAF-M) and *Campylobacter* in women (HSFA-W) were partially explained by high or low SFA intake. Recent studies have reported that HFD increases intestinal permeability and LPS translocation into the circulation, triggering systemic inflammation, oxidative stress and changes in gut microbiota composition (49, 50). Supporting our findings, the abundance of the genus *Anaerotruncus*, a butyrate producer, has been reported to increase in mice under HFD feeding (51, 52), and also in obese mice (53, 54) and humans (55). A study of older community-dwelling men reported a positive association between *Anaerotruncus* and a Western-type diet (56). Interestingly, this genus has been associated with metabolic disease, and was found to be more abundant in women than in men (24). In contrast to this latter study, we found that *Anaerotruncus* abundance is linked with SFA consumption in the entire population (both men and women) and also only when men were considered. In a study on intestinal barrier dysfunction, Gao et al. found that *Anaerotruncus* was related to intestinal permeability indices, LPS, and tight junction proteins in rats on HFD (50), suggesting its involvement in loss of barrier function and LPS production. *Campylobacter*, a genus that correlates with SFA intake in women in our study, comprises 20 species with the majority related to intestinal, systemic and periodontal infections (46). It has been reported that mice fed a diet rich in SFAs have an increased susceptibility to *Campylobacter* infection (57).

We found negative correlations between SFAs intake and abundance of *Anaerostipes* and *Ruminococcaceae* (*UCG-014*) in men. *Anaerostipes* abundance was inversely correlated with SFAs in the HSFA-M group. Similar to *Anaerotruncus, Anaerostipes* is a butyrate-producing genus that is associated with the gut microbiota of healthy individuals (58, 59). *Anaerostipes* has been reported to decrease in abundance in HFD-induced metabolic syndrome (60), and to increase after low-fat vegetarian diets (61) and fiber-rich diets (62) and also when the BMI decreases (63), in agreement with the data obtained in the present study. *Ruminococcaceae* have been shown to negatively correlate with metabolic disease in humans (64), and we observed that the genus *Ruminococcaceae* (*UCG-014*) negatively correlated with SFAs consumption. Zhao et al. reported similar results in rats, where this taxon significantly decreased in abundance after HFD feeding (65).

Men following an HSFA diet with a low fiber intake showed unfavorable anthropometric patterns, which could increase the risk of obesity and other related disorders. Also, *Campylobacter, Flavonifractor*, and *Erysipelotrichaceae*, all prevalent in the HSFA-W group, are associated with inflammation, indicating that high-fat intake increases the levels of inflammation, especially in women, which could be a reason for the high risk of obesity and colon cancer (43, 45, 46, 66). It is important to highlight that these bacterial taxa associated with different diseases have been found in healthy participants who consume high levels of SFAs. The bacterial taxa found in this population could be used as early predictors for the onset of some diseases and inflammatory disorders in the healthy population.

Focusing on the relevant functional categories highlighted by the PiCRUSt analysis, we found that the HSFA-W group showed a positive association with the presence of colicins, which is in accordance with a study reporting an enrichment of ferric enterobactin and colicins in women with high fat intake (67). Enterobactin presumably acts on transferrin to decrease iron assimilation. It is not yet known whether the high levels of dietary fat influence iron absorption, but it seems that women with obesity might have decreased iron assimilation (68). In men, SFAs consumption seems positively associated with HydG, a response regulator of hydrogenase 3 activity and also related to iron and other metal cofactors such as zinc (69). Iron is an essential nutrient for many bacteria, and iron supplementation has been reported to affect the composition of the gut microbiota (38). Iron deficiency in humans is the most common form of malnutrition, causing iron-deficiency anemia, and is likely due to poor iron absorption in the gut (68).

HSFA in men was associated with the presence of L-fucose permease and alpha-L-fucosidase, which increase the risk of *Campylobacter jejuni* infections (70). The HSFA-M group was also associated with a multidrug efflux system (71). Fucose is a product of the degradation of intestinal mucin. Prolonged high-fat diets induce low-grade chronic intestinal inflammation in mice, and diets high in SFAs are a risk factor for the development of human inflammatory bowel disease, resulting in mucin degradation and activation of fucose metabolism pathways. This likely increases the prevalence of *Campylobacter* spp. (70). LSFA in men seems to be associated with a better performance of the ABC-2 type transporters, which could favor the elimination of xenobiotics.

Overall, our study shows that the microbiota of healthy subjects following a diet rich in SFAs diet contains bacterial taxa that could be related to the development of certain diseases, especially obesity and other pro-inflammatory diseases in women, and correlates with the modification of anthropometrical patterns in men. Differences in gut microbiota according to sex suggest that women and men could be differentially influenced by HSFA diets. Moreover, consumption of SFAs was found to be associated with a low fiber intake; with their combined effect likely synergistic for promoting an unhealthy microbiota, which could influence the development of inflammatory and obesity-related diseases. The establishment of personalized diet interventions for preventing diseases will require a deeper understanding of how sex influences the response to diet and the development of chronic diseases, and how the gut microbiota can be a mediator between the two factors. Our study identifies bacterial taxa that could be considered as early predictors of disease onset in a healthy population.

## Data Availability Statement

The datasets presented in this study can be found in online repositories. The names of the repository/repositories and accession number(s) can be found below:

NCBI BioProject; Accession No. PRJNA647292.

## Ethics Statement

The studies involving human participants were reviewed and approved by Ethics Committee for Clinical Research of the Hospital Ramón y Cajal (Madrid, Spain) (CEIC 338-17). The patients/participants provided their written informed consent to participate in this study.

## Author Contributions

ML and MM performed the experiments. CB and MB analyzed the data. MB, CS, SM-L, ML, and RG-S wrote the paper. All authors contributed to the article and approved the submitted version.

## Conflict of Interest

The authors declare that the research was conducted in the absence of any commercial or financial relationships that could be construed as a potential conflict of interest.
