# Supplementary figures and images for: Microbiota Features Associated With a High-Fat/Low-Fiber Diet in Healthy Adults
Source: Front Nutr. 2020 Dec 18;7:583608. doi: 10.3389/fnut.2020.583608 (PMC7775391; doi:10.3389/fnut.2020.583608)

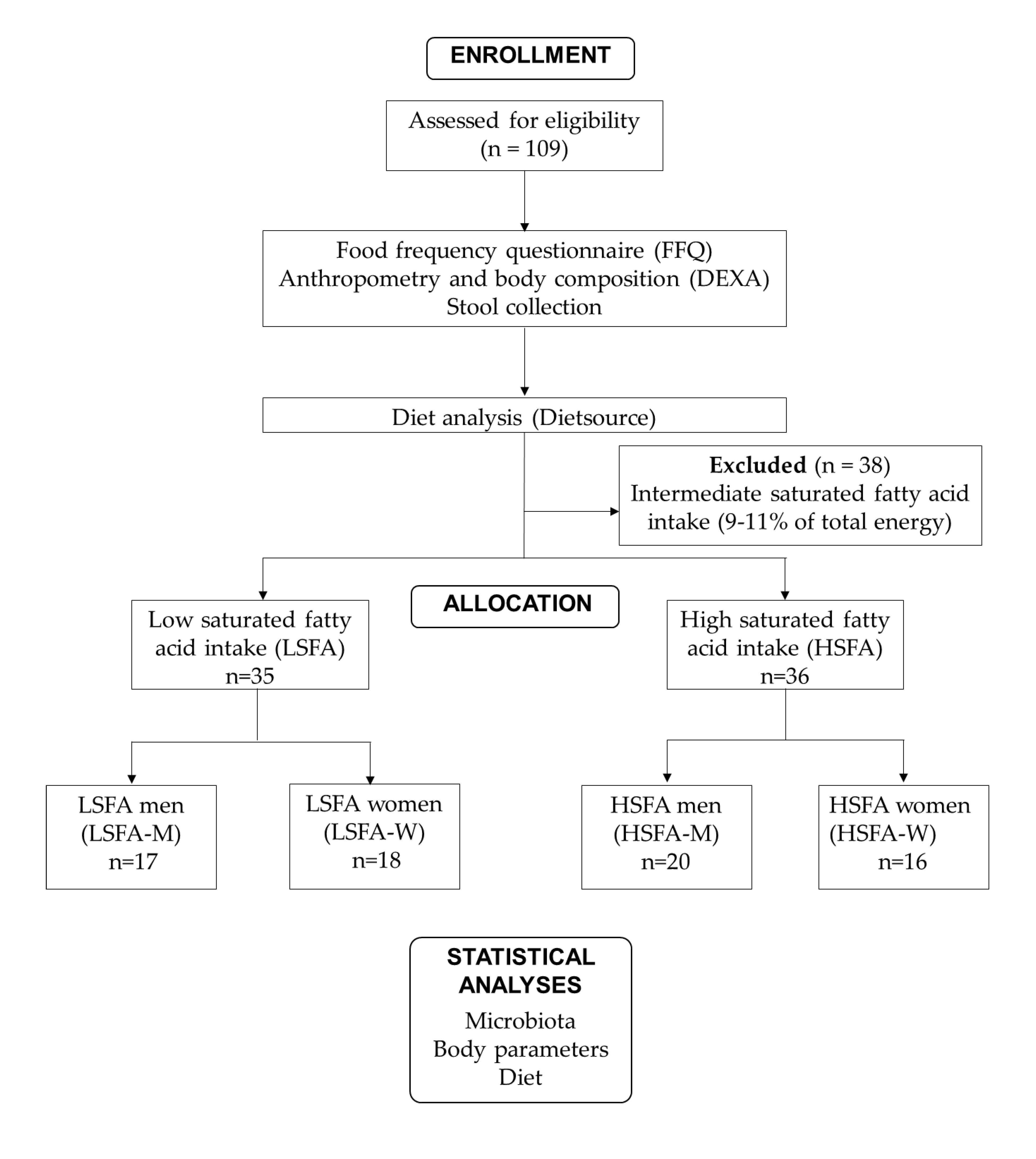

Supplement: Supplementary file 1 [file Image_1.TIF]

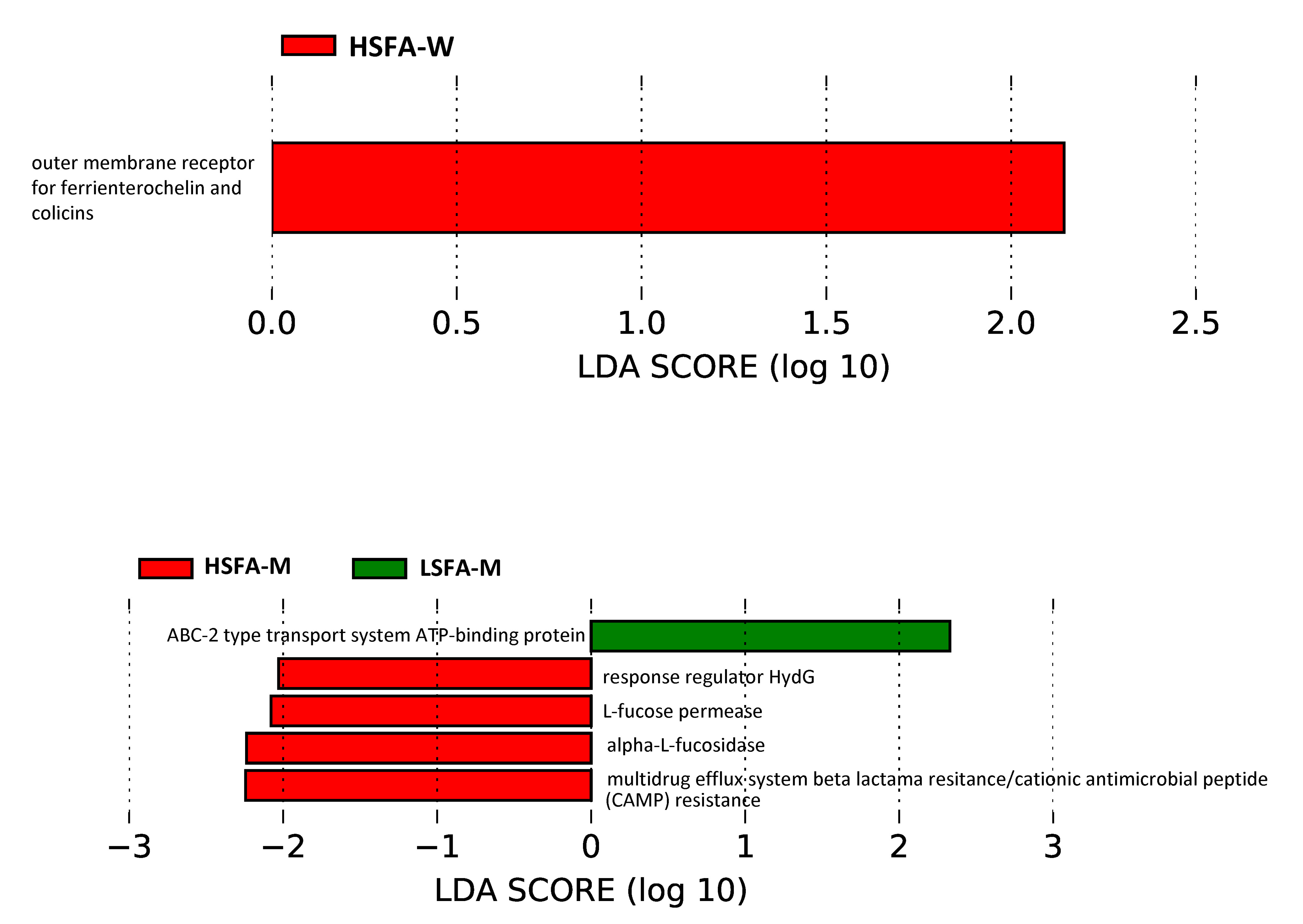

Supplement: Supplementary file 2 [file Image_2.TIF]
