# Supplementary material for: Microbiota Features Associated With a High-Fat/Low-Fiber Diet in Healthy Adults
Source: Front Nutr. 2020 Dec 18;7:583608. doi: 10.3389/fnut.2020.583608 (PMC7775391; doi:10.3389/fnut.2020.583608)
Supplement: Supplementary file 3 [file Table_1.DOCX]

Supplementary Material

**Table S1.** Food consumption

|  | **LSFA** | **HSFA** | **p** | **LSFA-M** | **HSFA-M** | **p** | **LSFA-W** | **HSFA-W** | **p** |
| --- | --- | --- | --- | --- | --- | --- | --- | --- | --- |
| Vegetables (s/d) | 3.28 ± 1.92 | 2.33 ± 1.22 | 0.016 | 2.45 ± 1.55 | 1.97 ± 1.03 | 0.28 | 4.01 ± 2.01 | 2.78 ± 1.33 | 0.05 |
| Fruits (s/d) | 3.17 ± 2.14 | 1.58 ± 1.09 | <0.001 | 3.18 ± 2.61 | 1.72 ± 1.30 | 0.04 | 3.17 ± 1.77 | 1.41 ± 0.77 | 0.00 |
| Potatoes | 0.10 ± 0.11 | 0.22 ± 0.22 | 0.006 | 0.09 ± 0.10 | 0.27 ± 0.26 | 0.009 | 0.11 ± 0.12 | 0.16 ± 0.16 | 0.32 |
| Sugars (s/d) | 0.57 ± 0.71 | 0.73 ± 0.79 | 0.365 | 0.45 ± 0.69 | 0.81 ± 0.95 | 0.22 | 0.70 ± 0.74 | 0.64 ± 0.54 | 0.77 |
| White meat (s/w) | 3.21 ± 2.37 | 2.53 ± 2.37 | 0.160 | 2.95 ± 1.54 | 2.36 ± 1.64 | 0.28 | 3.44 ± 3.01 | 2.73 ± 1.58 | 0.41 |
| Red meat (s/w) | 2.45 ± 1.31 | 3.01 ± 1.61 | 0.116 | 2.26 ± 1.23 | 3.46 ± 1.78 | 0.01 | 2.55 ± 1.39 | 2.44 ± 1.19 | 0.80 |
| Processed meat (s/w) | 1.62 ± 0.84 | 1.86 ± 0.97 | 0.268 | 1.33 ± 0.52 | 1.91 ± 0.94 | 0.02 | 1.89 ± 1.00 | 1.80 ± 1.03 | 0.78 |
| Dairy products (s/d) | 2.24 ± 1.23 | 2.39 ± 1.50 | 0.638 | 2.13 ± 1.11 | 2.39 ± 1.45 | 0.47 | 2.35 ± 1.39 | 2.39 ± 1.60 | 0.94 |
| Cereals (s/d) | 1.99 ± 1.01 | 2.32 ± 1.52 | 0.302 | 2.16 ± 1.19 | 2.70 ± 1.67 | 0.3 | 1.85 ± 0.88 | 1.89 ± 1.21 | 0.91 |
| Legumes (s/d) | 0.26 ± 0.19 | 0.19 ± 0.16 | 0.105 | 0.30 ± 0.21 | 0.19 ± 0.17 | 0.09 | 0.21 ± 0.17 | 0.19 ± 0.15 | 0.70 |
| Fish (s/w) | 6.10 ± 2.87 | 5.06 ± 1.79 | 0.074 | 6.00 ± 3.24 | 5.45 ± 1.97 | 0.55 | 6.24 ± 2.69 | 4.60 ± 1.49 | 0.03 |
| Eggs (s/w) | 2.85 ± 1.15 | 2.81 ± 1.41 | 0.880 | 3.13 ± 1.19 | 2.88 ± 1.54 | 0.60 | 2.61 ± 1.13 | 2.72 ± 1.29 | 0.80 |
| Nuts (s/w) | 2.83 ± 3.05 | 1.36 ± 1.38 | 0.013 | 2.84 ± 3.36 | 1.33 ± 1.33 | 0.13 | 2.70 ± 2.59 | 1.41 ± 1.48 | 0.08 |

s/d: servings per day; s/w: servings per week. LSFA-M: men with low saturated fatty acid intake. HSFA-M: men with high saturated fatty acid intake. LSFA-W: women with low saturated fatty acid intake. HSFA-W: women with high saturated fatty acid intake. Values are mean ± standard deviation.

**Table** **S2.** Baseline characteristics of subjects

|  | **LSFA-M** | **HSFA-M** | **p** | **LSFA-W** | **HSFA-W** | **p** |
| --- | --- | --- | --- | --- | --- | --- |
| Age (years) | 33.94 ± 9.08 | 33.00 ± 7.78 | 0.74 | 32.59 ± 9.14 | 30.38 ± 7.83 | 0.46 |
| Body mass (kg) | 71.26 ± 10.37 | 76.96 ± 7.58 | 0.79 | 59,18 ± 9.13 | 62.3 ± 8.51 | 0.32 |
| BMI (kg/m^2^) | 23.10 ± 2.50 | 25.09 ± 2.56 | **0.03** | 22.59 ± 2.67 | 23.50 ± 4.72 | 0.51 |
| BFP (%) | 19.32 ± 4.87 | 23.91 ± 5.53 | **0.01** | 31.45 ± 5.29 | 33.40 ± 6.18 | 0.39 |
| BFM (kg) | 69.26 ± 10.01 | 74.63 ± 7.31 | 0.08 | 58.57 ± 8.09 | 61.12 ± 8.46 | 0.43 |
| VAT (g) | 282.56 ± 121.65 | 426.26 ± 204.02 | **0.02** | 274.38 ± 153.64 | 270.29 ± 145.30 | 0.94 |
| AI (kg/m^2^) | 4.39 ± 1.36 | 5.90 ± 1.88 | **0.01** | 6.99 ± 1.76 | 7.31 ± 2.06 | 0.67 |
| MMI (kg/m^2^) | 17.20 ± 1.67 | 17.42 ± 1.32 | 0.66 | 14.23 ± 1.56 | 13.48 ± 1.22 | 0.17 |
| AppMMI (kg/m^2^) | 7.78 ± 0.95 | 7.95 ± 0.76 | 0.56 | 6.08 ± 0.92 | 5.85 ± 0.60 | 0.44 |

BMI: body mass index; BFP: body fat percentage; BFM: body fat mass; VAT: estimated visceral fat; AI: adiposity index; MMI: muscular mass index; AppMMI: appendicular muscular mass index. Values are mean ± standard deviation. LSFA-M: men with low saturated fatty acid intake. HSFA-M: men with high saturated fatty acid intake. LSFA-W: women with low saturated fatty acid intake. HSFA-W: women with high saturated fatty acid intake.

**Figure S1.** Study design flow-chart.

**Figure S2.** Predicted functional composition of metagenomes based on 16SrRNA gene sequencing data. LEfSe based on PICRUSt2 dataset revealed differentially enriched metabolic pathways associated with high and low intake of fatty acids for: (A) HSFA-W and LSFA-W (B) HSFA-M and LSFA-M.
